# Electrocardiogram frequency change by extracorporeal blood perfusion in a swine ventricular fibrillation model

**DOI:** 10.1186/1475-925X-12-123

**Published:** 2013-11-25

**Authors:** Jung Chan Lee, Gil Joon Suh, Hee Chan Kim

**Affiliations:** 1 Institute of Medical and Biological Engineering, Medical Research Center, Seoul National University, Yongon-dong 28, Jongro-gu, Seoul 110-744, Republic of Korea; 2 Department of Emergency Medicine, Seoul National University College of Medicine and Seoul National University Hospital, Yongon-dong 28, Jongro-gu, Seoul 110-744, Republic of Korea; 3 Department of Biomedical Engineering, Seoul National University College of Medicine and Seoul National University Hospital, Yongon-dong 28, Jongro-gu, Seoul 110-744, Republic of Korea

**Keywords:** Electrocardiogram (ECG), Frequency analysis, Extracorporeal cardiopulmonary resuscitation (ECPR), Ventricular fibrillation (VF)

## Abstract

**Background:**

Extracorporeal cardiopulmonary resuscitation (ECPR) refers to the application of extracorporeal blood circulation with oxygenation as a resuscitation tool. The objective of this study is to observe the frequency component changes in the electrocardiogram (ECG) by ECPR during prolonged ventricular fibrillation (VF).

**Methods:**

Six swine were prepared as a VF model. Extracorporeal blood circulation with a pulsatile blood pump and oxygenator was set up for the model. ECG signals were measured for 13 min during VF and analyzed using frequency analysis methods. The median frequency (MF), dominant frequency (DF), and amplitude spectrum area (AMSA) were calculated from a spectrogram obtained using short-time Fourier transform (STFT).

**Results:**

MF decreased from 11 Hz at the start to 9 Hz at 2 min after VF and then increased to 11 Hz at 4.5 min after VF. DF started at 7 Hz and increased to 11 Hz within the first min and decreased to 9 Hz at 2 min, then increased to 12 Hz at 4.5 min after VF. Both frequency components decreased gradually from 4.5 min until 10 min after VF. After the oxygenated blood perfusion was initiated, both MF and DF increased remarkably and exceeded 12 and 14 Hz, respectively. Similarly, AMSA decreased gradually for the first 10 min, but increased remarkably and varied beyond 13 mV∙Hz after the oxygenated blood supply started. Remarkable frequency increases in ECG due to the oxygenated blood perfusion during ECPR were observed in the swine VF model.

**Conclusions:**

The ECG frequency analysis during ECPR can give the resuscitation provider important information about the cardiac perfusion status and the appropriateness of the ECPR setup as well as the prediction of defibrillation success.

## Background

More than 50% of cardiac arrests are due to ventricular fibrillation (VF). Early defibrillation is the best treatment for short duration of VF. However, immediate defibrillation for prolonged VF with ischemia results in critical damage to the heart and increase of myocardial blood perfusion should be performed prior to countershock. Chest compression by cardiopulmonary resuscitation (CPR) procedure is an effective way to improve the myocardial blood flow during VF. To determine the suitable intervention for a specific time during VF and to predict the defibrillation success, various electrocardiogram (ECG) analysis methods have been studied. ECG during VF delivers much important information describing the fibrillating heart status. Analyzing the ECG during VF can be used to predict the defibrillation outcome [1]. Amplitude [2], frequency [3,4], bispectral analysis [5], amplitude spectrum area (AMSA) [6], wavelets [7,8], and N(α) histograms [9] obtained from an ECG signal have been used to estimate the duration of VF. Frequency analysis showed a high correlation between cardiac perfusion pressure and the return of spontaneous circulation (ROSC) [10]. Fibrillation frequency and myocardial blood flow are also highly correlated during VF [11]. In particular, several studies reported that an increase in fibrillation frequency during VF occurs due to the chest compressions in the CPR procedure [12,13]. These studies imply that a myocardial perfusion condition in fibrillating the heart could be estimated using the ECG frequency analysis.

Extracorporeal cardiopulmonary resuscitation (ECPR) refers to the application of extracorporeal blood circulation with oxygenation as a resuscitation tool. Since the first ECPR concept was proposed as early as the 1960s [14], many researches of ECRP were performed for in-hospital and out-of-hospital settings. Although the current resuscitation guidelines do not consider ECPR as a recommended practice particularly in out-of-hospital arrest due to its critical need for expertise [15,16], the improved outcomes of ECPR have been continuously reported for out-of-hospital events and pediatric applications [17-22].

Although ECRP aims to circulate the oxygenated blood through the body, in particular through the arrested heart, the change in the ECG frequency during ECPR has not been studied in detail. We assumed that a strong change of frequency component by an effective blood perfusion to the fibrillating heart would occur similar to the chest compression. We examined a VF animal model while delivering oxygenated blood to a fibrillating heart using a blood pump, and the ECG signals obtained during the VF were investigated using the frequency analysis methods.

## Methods

### Animal preparation and measurement

All experimental protocols involving animals were reviewed and approved by the Institutional Animal Care and Use Committee of the Seoul National University Hospital.

Six swine (body weight, 30 kg) were prepared. All animals had free access to food and water for 7 days. While they fasted 8 hours before experiments, they were given free access to water. The animals were anesthetized with an intramuscular injection of ketamine (6 mg/kg) and xylazine (1 mg/kg). Pancuronium (0.2 mL/kg) was administrated intravenously as a muscle relaxant. After endotracheal intubation, the animals were connected to a ventilator. The tidal volume was set at 15 ml/kg, and the ventilation rate was set at 22 breathes/min initially and adjusted to maintain end-tidal CO_2_ at 30–35 mmHg. Anesthesia was maintained with 1–2 L/min enflurane at a 1:1 ratio of O_2_/N_2_O. The right carotid artery was cannulated with a 5-Fr central venous catheter for blood pressure measurements and was rinsed with heparin saline. The internal jugular vein was cannulated for placement of a 5-Fr pacing electrode (St. Jude Medical Inc., St. Paul, MN, USA) to induce VF. A heparin dose of 250 IU/kg was administered for anticoagulation.

A 17-Fr arterial catheter was inserted into the abdominal aorta through the right femoral artery. A 15-Fr venous catheter was inserted through the right femoral vein, and its end tip was placed in the right atrium. The blood circuit, including the pump and the membrane oxygenator, was primed with heparinized saline. The draw line and return line of the circuit were connected to the venous and arterial catheters, respectively. T-PLS (BHK, Inc., Seoul, Korea) was employed as a blood pump, which was developed for a heart-lung machine and extracorporeal membrane oxygenation machine [23,24]. An Affinity Oxygenator (Medtronic, Inc., Minneapolis, MN, USA) and an Affinity Arterial Filter (Medtronic, Inc.) were used for membrane oxygenation and air-bubble filtering, respectively. Blood flow rates through the blood circuit were measured with an ultrasonic flow meter (Transonic System, Inc., Ithaca, NY, USA).

After all catheterizations had been completed, a tongue sensor was placed for monitoring oxygen saturation, and three standard lead II ECG electrodes were attached to the chest wall. Body temperature was monitored with a rectal thermistor.

**Figure 1:**
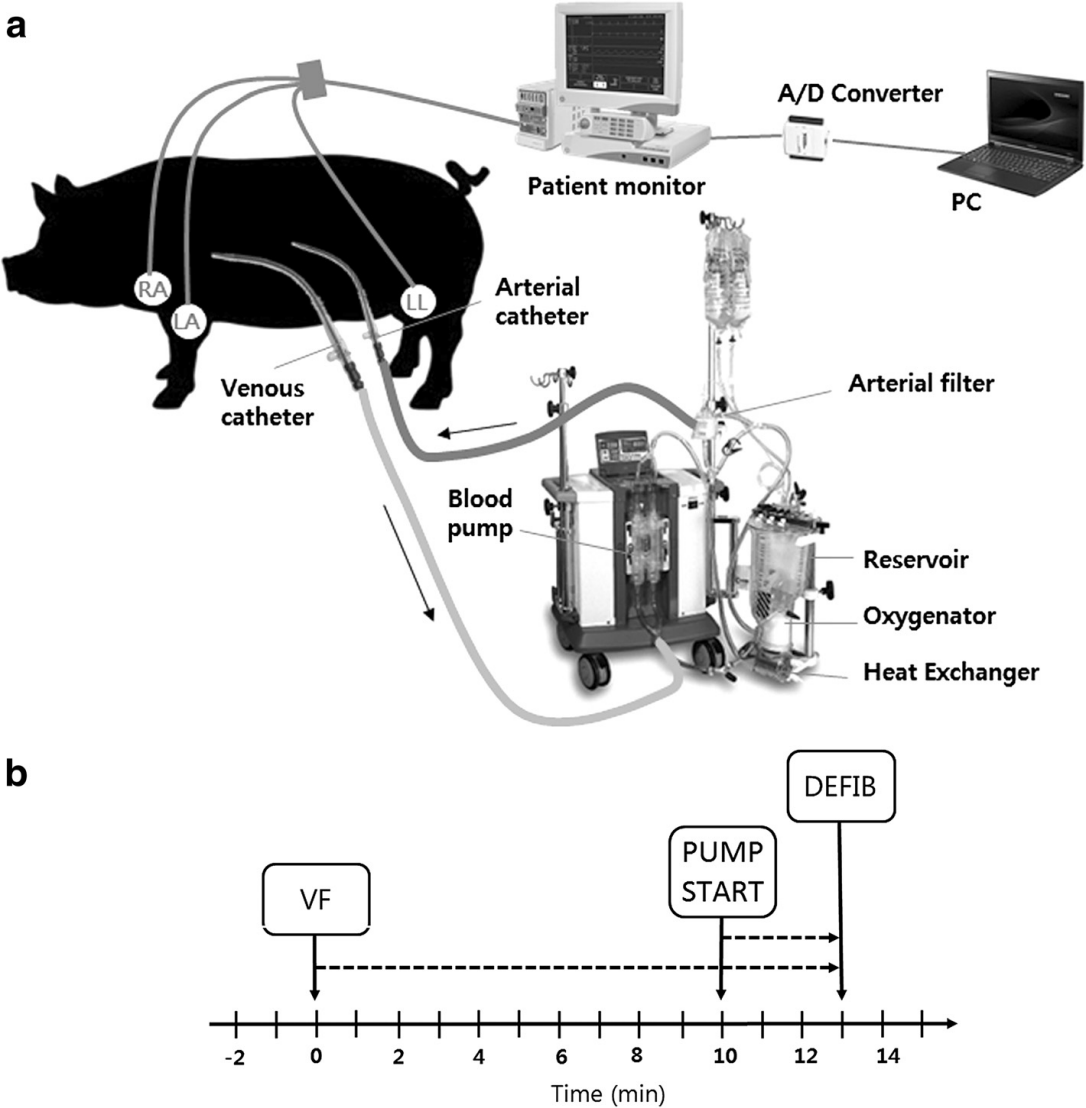
**Schematic diagram of (a) extracorporeal blood circulation and electrocardiogram (ECG) signal acquisition and (b) experimental timeline.** Blood pump was started at 10 min after ventricular fibrillation (VF) induction and defibrillation (DEFIB) was applied at 3 min after blood pump started. ECG waveforms were recorded for 13 min.

VF was induced with a 30–60 mA current for 20 sec via the pacing electrode using a custom-made electrical stimulator. The induction of VF was confirmed by both ECG waveform shape and a diastolic blood pressure below 25 mmHg. After successfully inducing VF, oxygen delivery was suspended by stopping the ventilator. After 10 minutes of untreated VF, blood perfusion was started to deliver oxygenated blood to the cardiac tissues. Defibrillation was applied 3 min after the blood pump onset. After a successful defibrillation, the ventilator resumed gas delivery. The blood flow rate by the pump was maintained at 2 L/min. Figure 1 shows the experimental timeline.

Oxygen saturation, ECG (lead II), body temperature, and blood pressure were monitored continuously during the experiment using a patient monitor (Solar 8000 M, GE Healthcare, Waukesha, WI, USA). ECG signals were transmitted to a PC via an A/D converter (NI USB-6008, National Instrument, Inc., Austin, TX, USA) and saved at a sampling rate of 300 Hz.

### Frequency analysis

For frequency analysis, ECG waveforms were preprocessed using a band-pass filter with a bandwidth of 4–50 Hz to reduce the low frequency noise (< 2 Hz) by pulsatile flow of the blood pump and the electrical interference of ambient noise (60 Hz).

Short-time Fourier transform (STFT) is a representative time-frequency analysis. STFT is also called as windowed Fourier transform. Segmenting the raw signal into a certain time intervals by using a window function, the Fourier transform is applied to each segment as the window function is slid along the time axis. As a result, STFT extracts information which indicates how the spectral content varies over the time. STFT of the time domain signal x(t) is given as:

(1) $$\mathrm{STFT}\left(\tau ,f\right)=\int_{-\infty }^{\infty }x\left(t\right)g\left(t-\tau \right)e^{-j2\mathrm{\pi ft}}\mathrm{dt}$$

where g(t − τ) is a sliding window function. While too wide window results in good frequency resolution and poor time resolution, too narrow window results in good time resolution and poor frequency resolution. In this study, the Hanning window was used as the window function, and its size was approximately 1.7 sec and contained 512 sample points. The Hanning window is known to be suitable to random signal and has good frequency resolution and reduced spectral leakage.

The spectrogram at time τ and frequency f can be acquired by computing the squared magnitude of STFT as follows:

(2) $$\mathrm{spectrogram}\left(\tau ,f\right)={\left|\int_{-\infty }^{\infty }x\left(t\right)g\left(t-\tau \right)e^{-j2\mathrm{\pi ft}}\mathrm{dt}\right|}^{2}$$

Median frequency (MF) indicates the frequency of the spectral mass center of the power spectrum and is calculated as follows:

(3) $$\mathrm{MF}\left(\tau \right)=\frac{\sum_{i=4}^{50}f_{i}\left(\tau \right)\times p_{i}\left(\tau \right)}{\sum_{i=4}^{50}p_{i}\left(\tau \right)}$$

where f_*i*_(τ) is the frequency component at time τ, and p_*i*_ is the power component at frequency f_*i*_(τ) [3].

Dominant frequency (DF) is the peak power frequency, defined as follows:

(4) $$\mathrm{DF}\left(\tau \right)=f_{\max\left(p\right)}\left(\tau \right)$$

where f_max(p)_(τ) is the frequency with the maximum power component at time τ [25].

AMSA indicates the area of the amplitude spectrum curve at a point of time and is calculated as follows:

(5) $$\mathrm{AMSA}\left(\tau \right)=\sum_{i=4}^{50}A_{i}\left(\tau \right)\times f_{i}\left(\tau \right)$$

where A_i_(*τ*) is the amplitude (mV) at frequency f_i_(*τ*) at time *τ*, and the frequency band is between 4 and 50 Hz [6,26].

All calculations were performed using the numerical computing software, Matlab (MathWorks, Inc., Natick, MA, USA). The STFT results were demonstrated by spectrogram. MF, DF, and AMSA were presented as the mean and standard deviations.

## Results

All procedures and signal measurements were successfully accomplished without any problems in all animals. Each defibrillation trial after a 3-min ECPR was successful.

**Figure 2:**
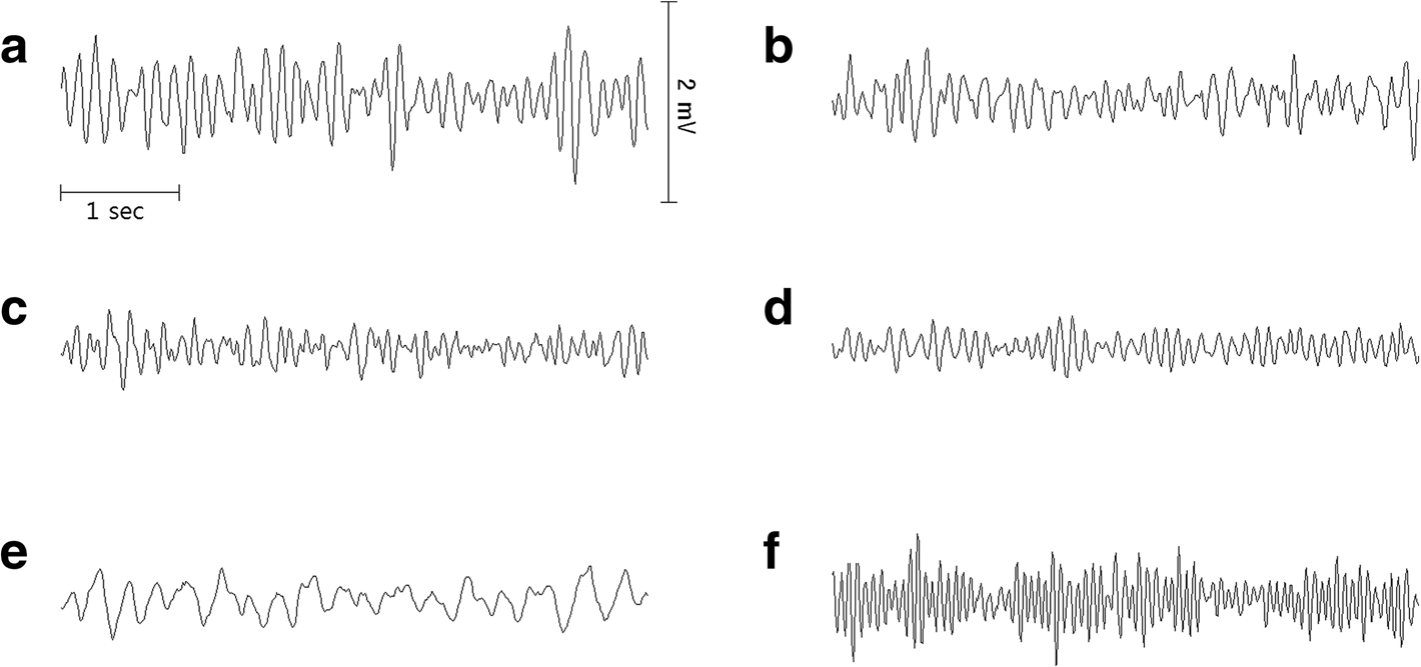
**Representative 5-sec electrocardiogram (ECG) waveforms during ventricular fibrillation (VF). (a)** 1 min, **(b)** 4 min, **(c)** 6 min, **(d)** 8 min, **(e)** 10 min (immediately after the blood pump start), and **(f)** 12 min after VF induction.

**Figure 3:**
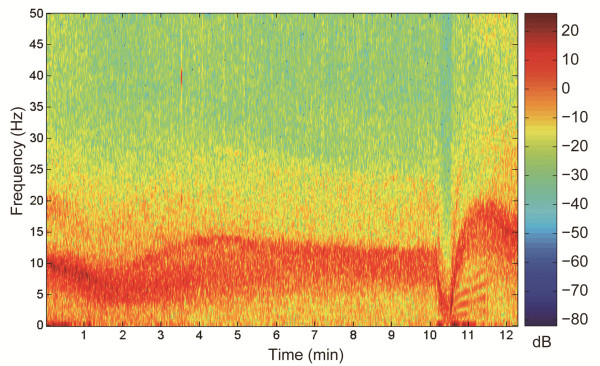
**A spectrogram analysis from electrocardiogram (ECG) signal obtained during ventricular fibrillation (VF).** The Hanning window function was used with a size of 1.7 sec with 512 sample points. Supplying oxygenated blood started at 10 min after VF induction. To show the original characteristic of the ECG signal, this spectrogram is not preprocessed by a 4–50 Hz band-pass filter. As a result, remarkable harmonic spectral lines with a strong low frequency spectrum observed for a minute due to a mechanical artifact by a pulsatile blood perfusion after ECPR was initiated. The color bar indicates the power in dB with red representing high power and blue low power.

Figure 2 shows the representative 5-sec ECG waveforms successively obtained from the same swine VF model. Figure 3 shows a representative typical spectrogram calculated by STFT during VF, which was not preprocessed by a 4–50 Hz band-pass filter. After VF induction, a strong power band appeared at approximately 10 Hz with a downward trend to 6–7 Hz until 2 min. It then rebounded and peaked beyond 10 Hz until 4.5 min. After 5 min, the band decreased slightly until 10 min. The strong frequency band disappeared immediately after the oxygenated blood was supplied. However, approximately 30 sec later, a strong band developed again from a very low frequency region and increased remarkably above 15 Hz within 2 min. Simultaneously, several harmonic spectral lines were observed with a strong low frequency spectrum for 1 minute.

**Figure 4:**
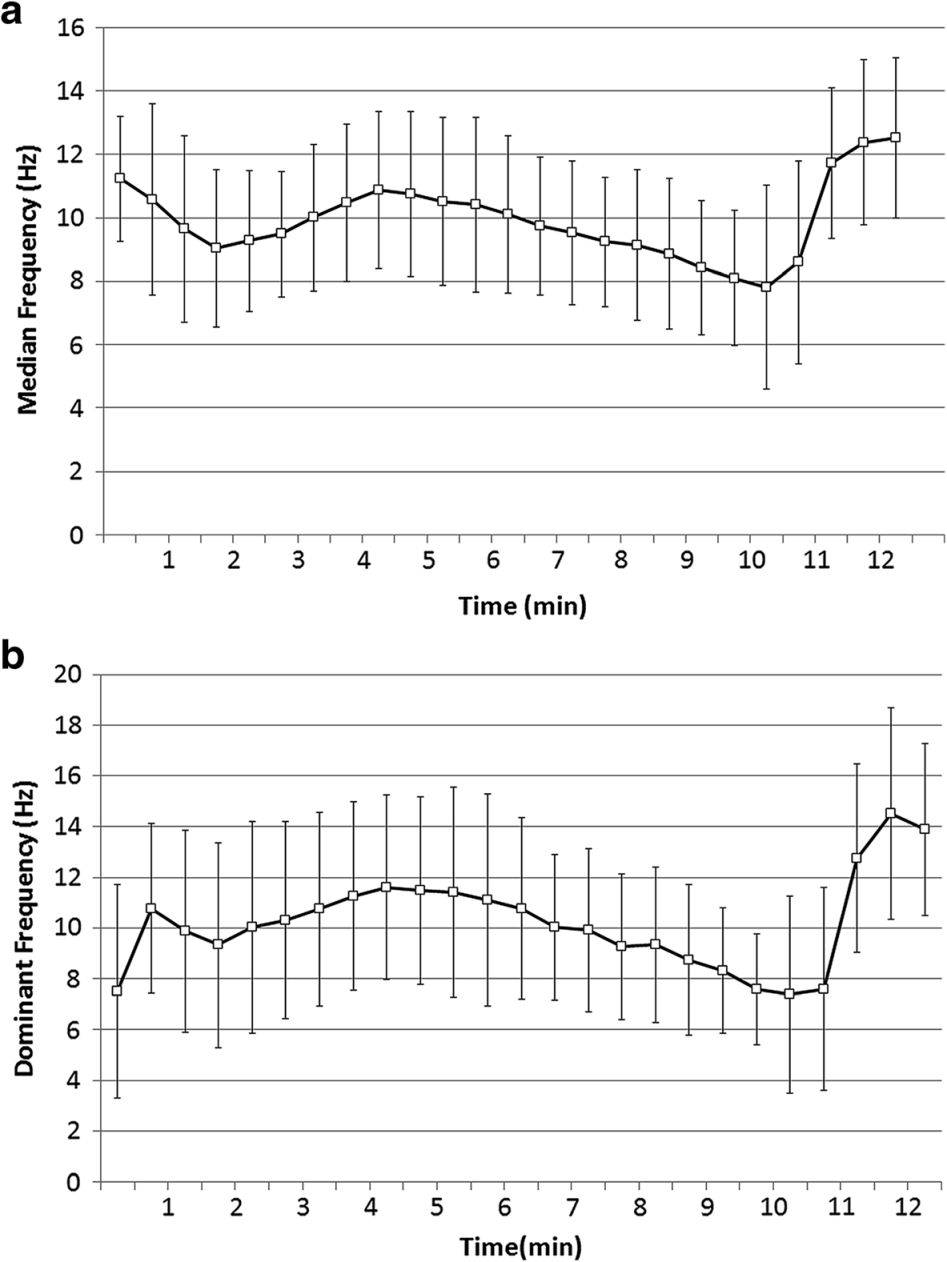
**Electrocardiogram (ECG) frequencies calculated every 30 sec during ventricular fibrillation (VF). (a)** Median frequency (MF) and **(b)** dominant frequency (DF). Oxygenated blood was supplied to a fibrillating heart after approximately 10 min. Squares and error bars indicate mean values and standard deviation, respectively.

**Figure 5:**
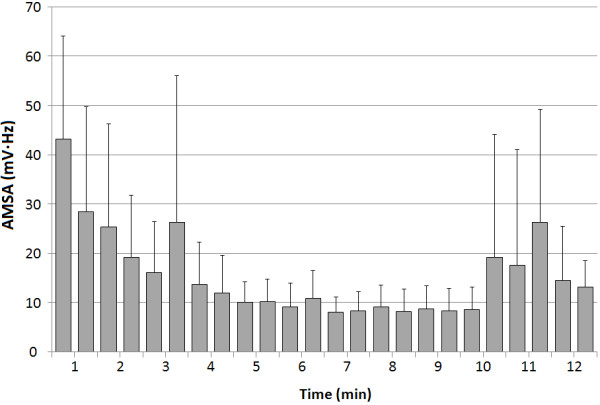
**Mean amplitude spectrum area (AMSA, mV∙Hz) calculated every 30 sec during ventricular fibrillation (VF).** Error bars indicate the standard deviation.

The mean MF value was 11 Hz at the start during VF, which decreased to 9 Hz until 2 min, and then rebounded to 11 Hz until 4.5 min (Figure 4.a). However, DF presented a different initial trend to that of MF (Figure 4.b). DF started at 7 Hz and increased to 11 Hz within 1 min, decreased to 9 Hz until 2 min, then increased again to 12 Hz until 4.5 min. After the start of the oxygenated blood supply, both MF and DF increased significantly after a short slight descent and exceeded 12 Hz and 14 Hz, respectively. Similarly, the mean AMSA decreased gradually until 10 min but then increased remarkably after the start of the blood pump (Figure 5).

## Discussion

The ECG has been widely used to observe cardiac physiology and to predict possible heart arrest. The amplitude of the ECG waveform during VF is a very effective indicator to predict survival outcome, and it steadily decreases during VF with increasing arrest time [25]. Another study reported that VF frequency change has been applied to estimate cardiac arrest duration by analyzing the trend in frequency change [26]. Both MF and DF can increase by performing CPR, and the MF, in particular, is related to the myocardial blood flow rate [11]. Furthermore, CPR prior to defibrillation elevates MF from 8.8 Hz to 15.1 Hz and improves the outcome of the ROSC significantly [12]. In this study, we showed that the oxygenated blood supply after 10 min of VF elevated MF from 8 Hz to 12 Hz and DF from 9 Hz to 14 Hz in swine VF models, suggesting that ECPR during VF improved the myocardial blood flow rate and helped the recovery of cardiac function.

The three-phase time-sensitive model suggested by Weisfeldt *et al.*[27] is helpful to understand the successive features of cardiac arrest and to perform appropriate therapy for each phase. The three phases of the cardiac arrest model are: (1) the electrical phase, which spans approximately 4 min from the start of cardiac arrest, (2) the circulatory phase from approximately 4 to 10 min, and (3) the metabolic phase extending beyond approximately 10 min after cardiac arrest. Early defibrillation is the most effective intervention during the electrical phase. However, the most lifesaving therapy during the circulatory phase is performing CPR to provide oxygen delivery followed by defibrillation. During the metabolic phase, the effectiveness of both immediate defibrillation and CPR prior to defibrillation is poor, but hypothermia is an effective method to prevent ischemic cell damage in tissues. In this study, the MF and DF variations observed during ECPR could be explained by the three-phase time-sensitive model. During the electrical phase (0–4 min), both the MF and DF decreased slightly and then increased to a peak. Throughout the circulatory phase (4–10 min), both frequencies decreased gradually. After initiation of ECRP (10 min), the frequencies increased again after a short descent as the oxygenated blood was delivered to the heart and reached another peak. The descent might be due to an instantaneous delivery of the deoxygenated blood which was previously located in the aorta and the priming solution in the blood circuit. Several harmonic spectral lines with a strong low frequency spectrum observed for a minute after ECPR was initiated. The low frequency artifacts diminished after MF and DF reached the peaks. These spectral harmonics might be due to a mechanical artifact by ECPR perfusion.

Similarly, AMSA decreased gradually after VF induction until ECPR was started and rebounded from a bottom plateau around 8 mV∙Hz up to about 26 mV∙Hz after the blood pump started (Figure 5). The typical AMSA values for swine or human models has been known as a range between 5–30 mV∙Hz, and the values above 13–21 mV∙Hz during arrest are a strong predictor of survival [28,29]. In the present study, mean AMSA increased after the oxygenated blood supply started and varied beyond 13 mV∙Hz until the defibrillation was performed. This increase of AMSA might make every trial of defibrillation successful.

Some limitations to this experimental study should be noted. First, we did not assess the outcome of defibrillation success or ROSC. Although all trials of defibrillation after ECPR were successful, the effectiveness of ECPR for improving ROSC is still unproven through this study. However, clinical reports comparing the effectiveness of ECPR and conventional CPR are currently being published [30,31]. Second, coronary blood flow was not simultaneously measured with the ECG during ECPR support, although the measurement method for the parameters was well known. We were afraid that the invasive measurement might distort the ECG signal during VF and ECPR. However, some studies reported a correlation between fibrillation frequency and coronary perfusion or myocardial blood flow during manual CPR in a swine model [12,13].

The results we obtained in the present study imply that the simultaneous ECG frequency analysis during ECPR gives the resuscitation provider important information about the cardiac perfusion status. When a problem occurred with cannulation or extracorporeal circuit setup, the oxygenated blood would not be properly delivered to the fibrillating heart even if the extracorporeal blood pump started on time. The resuscitation provider could timely recognize an occurrence of the critical problem by monitoring the ECG frequency change after ECPR support started, and an immediate step would be taken to solve the problem.

## Conclusions

We have measured the ECG signals in a swine VF model and performed frequency analyses to observe the change in fibrillation frequency by supplying oxygenated blood to a fibrillating heart. Remarkable frequency increases in ECG due to the oxygenated blood perfusion during ECPR were observed. The ECG frequency analysis during ECPR can give the resuscitation provider important information about the cardiac perfusion status and the appropriateness of the ECPR setup as well as the prediction of defibrillation success.

## Competing interests

The authors declare that they have no competing interests.

## Authors’ contributions

The animal experimental study was planned and performed by JCL with support by GJS. The signal acquisition and frequency analysis were performed by JCL with support by HCK. The manuscript was written by JCL, and revised by JCL, GJS and HCK. All authors approved the final version of the manuscript.
